# Safety, tolerability and preliminary efficacy of ALMB-0166 in patients with acute spine cord injury

**DOI:** 10.1093/braincomms/fcag275

**Published:** 2026-07-25

**Authors:** Jinqian Liang, Wei Chen, Fang Zhou, Wenge Wang, Weixiang Kong, Pingping Chen, Juehua Jing, Haijiao Mao, Youliang Hao, Qingxi Wang, Chao Li, Deliang Yin, Miao Jin, Shaonan Ni, Wen You, Yanfeng Zhang, Guixing Qiu

**Affiliations:** Department of Orthopedics, Peking Union Medical College Hospital, Beijing 100730, P.R. China; Department of Orthopedic Surgery, Hebei Medical University Third Hospital, Shijiazhuang 050051, P.R. China; Department of Orthopedics, Peking University Third Hospital, Beijing 100191, P.R. China; Department of Orthopedics, Linfen Central Hospital, Linfen 041000, P.R. China; Phase I Clinical Research Center, Jining No.1 People's Hospital, Jining 272002, P.R. China; Department of Neurosurgery, Union Hospital Affiliated to Fujian Medical University, Fuzhou 350001, P.R. China; Department of Orthopedics, The Second Affiliated Hospital of Anhui Medical University, Hefei 230601, P.R. China; Department of Orthopedics, The First Affiliated Hospital of Ningbo University, Ningbo 315000, P.R. China; Department of Orthopedics, Peking University Third Hospital, Beijing 100191, P.R. China; Clinical Development Division, CSPC Pharmaceutical Group Co., Ltd., Shijiazhuang 050031, P.R. China; Clinical Development Division, CSPC Pharmaceutical Group Co., Ltd., Shijiazhuang 050031, P.R. China; Clinical Development Division, CSPC Pharmaceutical Group Co., Ltd., Shijiazhuang 050031, P.R. China; Clinical Development Division, CSPC Pharmaceutical Group Co., Ltd., Shijiazhuang 050031, P.R. China; Clinical Development Division, CSPC Pharmaceutical Group Co., Ltd., Shijiazhuang 050031, P.R. China; Clinical Development Division, CSPC Pharmaceutical Group Co., Ltd., Shijiazhuang 050031, P.R. China; Research & Development, AlaMab Therapeutics Inc., Princeton, NJ 08540, USA; Department of Orthopedics, Peking Union Medical College Hospital, Beijing 100730, P.R. China

**Keywords:** ALMB-0166, acute spinal cord injury, safety, tolerability, efficacy

## Abstract

ALMB-0166 is a first-in-class humanized monoclonal antibody that blocks connexin-43 hemichannels on spinal cord astrocytes. By reducing astrocyte and microglia activation, attenuating axonal degeneration and preventing the release of ATP, glutamate, ions and other molecules that drive excitotoxicity, inflammation and metabolic stress, ALMB-0166 mitigates secondary injury following spinal cord injury (SCI) and promotes neurological recovery. The objective of this study was to evaluate the safety, tolerability, pharmacokinetics (PKs) and efficacy of ALMB-0166 in patients with acute SCI. Eligible patients had acute SCI at or below the C3 level, with an American Spinal Injury Association Impairment Scale (AIS) grade of B or C, and were randomized within 72 h after injury. Patients received a single intravenous dose of ALMB-0166 (200, 600, 1200, 2400 or 4800 mg) or placebo, along with best supportive care. The primary outcome was safety. Secondary outcomes included PKs, immunogenicity and efficacy. A total of 25 patients with C3–C7 SCI were enrolled, of whom 24 received treatment (17 with ALMB-0166 and 7 with placebo). Treatment-emergent adverse events (TEAEs) occurred in 94.1% (16/17) of patients in the ALMB-0166 group and 100% (7/7) of those in the placebo group. Grade ≥3 TEAEs were reported in 17.6% (3/17) of the ALMB-0166 group and 42.9% (3/7) of the placebo group. Treatment-related AEs (TRAEs) occurred in six patients (35.3%) with ALMB-0166 and two patients (28.6%) with placebo; all TRAEs were Grade 1. No deaths occurred. ALMB-0166 exhibited essentially linear PKs over the dose range of 200–4800 mg, with concentrations increasing gradually from the start of infusion, peaking near the end of infusion, and then declining slowly. Compared with placebo, ALMB-0166 treatment was associated with improvement in motor function, sensory function, AIS and pain. Specifically, two patients in the ALMB-0166 group recovered from AIS Grade C to Grade E, whereas no patient in the placebo group recovered to Grade E. Notably, the 1200 and 2400 mg dose groups showed superior efficacy in sensory and motor measures; pooled data from these two groups showed greater improvements in both sensory and motor scores than those in the placebo group. In conclusion, ALMB-0166 demonstrated a manageable safety profile and improved neurological recovery in patients with acute SCI.

## Teaser

Liang *et al*. report that ALMB-0166, a first-in-class anti-connexin-43 antibody, was safe and well-tolerated in a Phase I/II trial of 24 patients with acute spinal cord injury, with preliminary improvements in sensory and motor function and neurological grade conversion, especially at pooled 1200/2400 mg doses versus placebo.

## Graphical Abstract

**Graphical Abstract:**
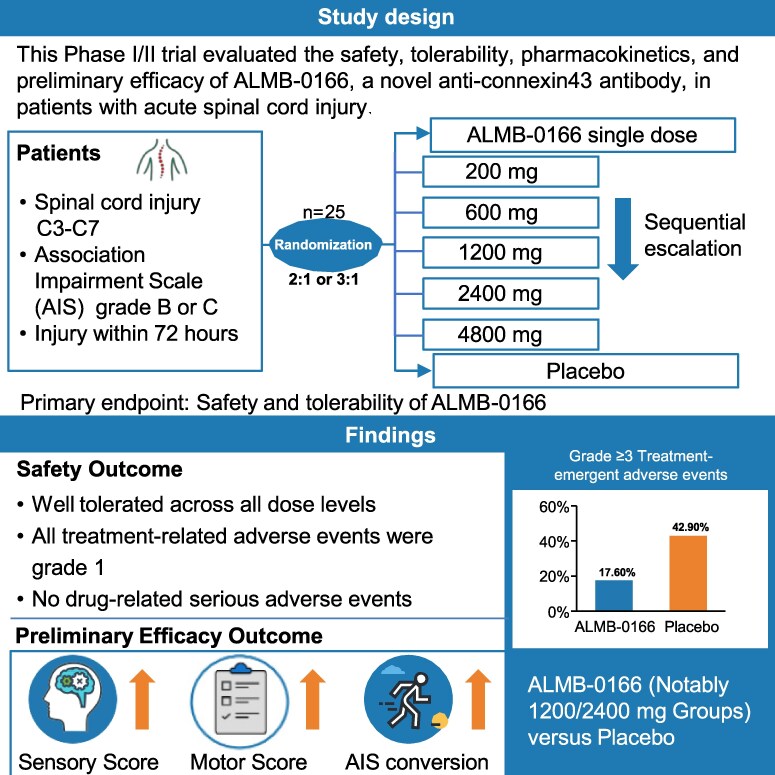

## Introduction

Spinal cord injury (SCI) is a devastating condition that results in a sudden loss of motor, sensory and autonomic functions below the level of injury.^1,2^ This life-altering neurological condition not only affects patients but also imposes a significant socioeconomic burden on their caregivers.^3^ More than 90% of SCI cases are traumatic in aetiology, caused by incidents such as traffic accidents, violence, sports or falls.^4,5^ Acute SCI results from mechanical trauma to the spinal cord, which may cause contusion, compression (by blood or bone fragments), partial tear or complete transection. The harmful neurological consequences of SCI arise from both primary mechanical injury and secondary injury process, including glutamate excitotoxicity, oxidative stress, ionic and metabolic imbalances, inflammation and ischaemia. These secondary processes lead to necrosis and cell death. The mechanisms of secondary injury play an important role in the loss of neurological function following trauma.^6^ Neuroinflammatory responses after injury trigger astrocyte activation and glial scar formation, creating a non-permissive environment that prevents axonal regeneration and thereby limits functional recovery.^3,7^ Acute SCI is a medical emergency requiring immediate intervention. The severity of symptoms (e.g. weakness, paralysis, sensory loss) depends on the degree of spinal cord damage and the anatomical level of injury.

In recent years, significant progress has been achieved in the medical management of SCI, remarkably improving the diagnosis rate, disease stabilization rate, survival rate and patients’ quality of life. However, despite these advances, the prognosis of neurological recovery in patients with acute SCI remains dismal, highlighting a critical gap in current treatment strategies.

Current therapeutic approaches for acute SCI are predominantly focused on mitigation rather than cure. Standard treatment protocols, including spinal cord decompression or stabilization surgery and comprehensive rehabilitation, aim to minimize secondary damage, facilitate neurological recovery and enhance patients’ quality of life.^2^ To limit secondary damage, various pharmacological interventions have been explored, such as non-steroidal anti-inflammatory drugs, minocycline, cyclosporine A and the corticosteroid methylprednisolone.^8^ These medications also address associated symptoms, including pain, urinary tract dysfunction, deep vein thrombosis and psychological disorders. Nevertheless, the downstream and potential off-target effects of these drugs on neurological recovery remain poorly understood. To date, little is known about the degree to which common drugs used in the management of acute SCI have downstream and unintended effects that modify neurological recovery.^9^ Early high-dose methylprednisolone therapy was once thought to have a positive effect on neural repair in the acute phase of SCI. However, its efficacy is constrained by a narrow therapeutic window (administration must occur within 8 h) and limited benefits, with only a subset of patients experiencing improved motor function. Moreover, this treatment is associated with severe side-effects, including immunosuppression and gastrointestinal complications.^10^ Therefore, methylprednisolone has not been recommended in the guidelines of the American Association of Neurosurgeons (AANS) and the Congress of Neurological Surgeons since 2013. This underscores the urgent need for innovative therapies that target the underlying pathological mechanisms to promote functional recovery.

Over the past decade, numerous therapies have been investigated in clinical trials, bringing new hope for patients with SCI. These emerging approaches aim to overcome the limitations of current treatments and represent a significant step towards developing more effective interventions for this devastating condition. However, effective therapies that improve neurological and functional recovery remain lacking. At present, no pharmacological interventions are available to enhance the extent to which a person recovers neurologically or functionally from acute SCI.^9^

Secondary injury cascades, particularly neuroinflammation-driven ‘bystander damage’, are mediated by aberrant crosstalk between astrocytes and microglia.^11^ Within this process, connexin43 (Cx43), the most abundant hemichannel protein in spinal cord astrocytes, plays a pivotal role.^12^ Emerging evidence indicates that Cx43-mediated astrocyte–microglia crosstalk, through the release of adenosine triphosphate (ATP) and other gliotransmitters, modulates the activation of pro-inflammatory signalling pathways (such as NF-κB) and microglial polarization, thereby amplifying neuroinflammation and secondary damage in central nervous system injury.^7^ It has been demonstrated that alterations in the expression levels of Cx43 are involved in inflammatory processes, such as those occurring during skin wounding and neuroinflammation.^13^ After events such as stroke, epilepsy, ischaemia, optic nerve injury and SCI, an elevation in Cx43 has been noted. In these cases, hemichannel opening occurs, leading to enhanced secondary damage through the inflammatory response. Modulating Cx43 has been recognized as a potential target for safeguarding and promoting repair in the context of neuroinflammation.^13^ Genetic and pharmacological inhibition of Cx43 has demonstrated neuroprotection in SCI models. Conditional deletion of astrocytic Cx43 in mice significantly reduces ATP release after injury. This reduction leads to multiple beneficial outcomes, including a 50% decrease in lesion volume, attenuation of glial scar formation, reduction in astrogliosis and activated microglia and improvement in motor function.^14^ Previous studies have demonstrated that Cx43 hemichannels play a critical role in injury spread after spinal cord trauma, suggesting a potential treatment target in SCI.^13^

ALMB-0166 is a novel humanized monoclonal antibody. By inhibiting Cx43 hemichannels in spinal cord astrocytes, it reduces astrocytes and microglia activation after acute SCI, thereby decreasing the release of molecules such as ATP, glutamate, ions and other substances that cause excitotoxicity, inflammation and metabolic stress.^15^ Preclinical cell tests have shown that ALMB-0166 effectively inhibits pathological opening of Cx43 hemichannels without affecting normal gap junction function. ALMB-0166 binds specifically to astrocytes from mice, rats, monkeys and humans. Binding of ALMB-0166 to its target suppresses the pathological opening of Cx43 hemichannels. In mice, ALMB-0166 administration inhibits secondary injury after SCI surgery, reduces the size of the residual SCI lesions and the proliferation of surrounding astrocytes and significantly improves motor function recovery in injured animals (unpublished data). Therefore, ALMB-0166 has the potential to inhibit secondary injury after SCI and promote neurological and motor functional recovery.

In this study, we aimed to evaluate the safety, tolerability, pharmacokinetic (PK) profile and preliminary efficacy of ALMB-0166 in patients with acute SCI.

## Materials and methods

### Study design

This was a multicentre, randomized, double-blind, placebo-controlled, single-ascending dose Phase I/II study evaluating the safety, tolerability, PK profile and preliminary efficacy of ALMB-0166 in patients with acute SCI. Five dose groups were predefined: 200 (or 3 mg/kg), 600, 1200, 2400 and 4800 mg. The study used an Interactive Web Response System (IWRS) and implemented block randomization stratified by dose group, performed by the statistician. Six patients were enrolled in each of the 200 and 600 mg dose groups and randomly assigned to the test and control groups in a 2:1 ratio; four patients were enrolled in each of the 1200, 2400 and 4800 mg groups and randomly assigned in a 3:1 ratio. Patients in the test and control groups received either a single intravenous dose of the test drug or placebo; all patients received optimal supportive care. Throughout the study, any necessary concomitant medications (including analgesics) or treatments were permitted, with the exception of ganglioside drugs.

Each patient was closely observed for 72 h after dosing and followed up on Days 7, 14, 28 and 56 post-dosing. If any adverse event (AE) occurred, the investigator had discretion to conduct an unscheduled visit or to extend the follow-up. A safety review committee (SRC) was established for the study, and the SRC was responsible for deciding whether to proceed to the next dose group after evaluating all available safety data for a given dose group, or whether to add other unplanned dose groups. If the safety and tolerability of a dose group remained favourable up to the highest preset dose group, the SRC could decide to initiate a higher dose.

This study was approved by the Independent Ethics Committee at each study site and was conducted in accordance with the Good Clinical Practice guidelines and the Declaration of Helsinki. Written informed consent was obtained from all patients before study initiation. This trial was registered at ClinicalTrials.gov (NCT05524103).

### Patients

Eligible patients were aged 18–75 years (inclusive) and had an American Spinal Injury Association Impairment Scale (AIS) Grade B or C according to the International Standards for Neurological Classification of Spinal Cord Injury (ISNCSCI; revised 2000, formerly ASIA standards). Patients had SCI at or below the C3 segment and were scheduled to undergo spinal surgery within 72 h after the initial injury. Main exclusion criteria included penetrating SCI or complete spinal cord transection; accompanying traumatic brain injury with visible structural lesions or diagnostic images (e.g. intracranial haemorrhage); contraindications to lumbar puncture; or a history of serious diseases involving other organ systems (e.g. heart, lungs, liver or kidneys) that, in the investigator’s judgement, rendered the patient unsuitable for trial participation. Patients with acute and chronic diseases that had caused neurological deficits were also excluded.

### Assessments

The primary endpoint was to assess the safety and tolerability of a single dose of ALMB-0166. Safety assessment included physical examination, vital signs, laboratory tests, electrocardiogram and incidence of AEs. The main laboratory assessments comprised complete blood count, serum biochemistry (liver/renal function, electrolytes, glucose, lipids), coagulation (prothrombin time, activated partial thromboplastin time, international normalized ratio), urinalysis, cardiac troponin I/T, cerebrospinal fluid (CSF) analysis and other tests as clinically indicated. Routine haematology, biochemistry, coagulation, urinalysis and troponin were performed at screening, 12 h post-dose (Day 1), and on Days 2, 3, 4, 7, 14, 28 and 56. CSF analysis and additional haematology tests were conducted at screening, 12 h post-dose (Day 1), Day 3 and Day 7, with collection frequency adjustable at the investigator’s discretion based on patient safety. AEs were monitored from the time of signing the informed consent form until D56 visit. AEs were coded using the Medical Dictionary for Regulatory Activities (MedDRA, version 25.1 or higher) and graded according to the National Cancer Institute Common Terminology Criteria for Adverse Events (NCI-CTCAE, version 5.0). The secondary endpoints consisted of PK profile, immunogenicity and efficacy. Plasma samples were obtained from patients at pre-dose, immediately after dose and 2, 5, 9, 24, 48 and 72 h after the start of infusion, as well as on Days 7, 14, 28 and 56. Plasma concentrations of ALMB-0166 were determined using a validated electrochemiluminescence assay. Plasma samples for immunogenicity testing were collected pre-dose and on Days 14, 28 and 56. The immunogenicity of ALMB-0166 was assessed by the incidence of anti-drug antibody (ADA). Efficacy was assessed at scheduled intervals using the ISNCSCI standards (2000 revision), which includes motor scores, sensory scores, neurological level of injury and AIS grade. Pain was evaluated concurrently using the Visual Analogue Scale (VAS). These examinations were performed at baseline and on Days 2, 3, 4, 7, 14, 28 and 56 after treatment. Study personnel who received appropriate training in ISNCSCI examination performed all patient evaluations and were blinded to treatment allocation throughout the study. Improvement from baseline was calculated by subtracting baseline scores from each subsequent score.

### Statistical analysis

The sample size was not determined based on formal statistical power calculations. It was determined empirically to allow initial evaluation of safety and tolerability while minimizing exposure in this vulnerable population. This approach is supported by prior first-in-human data in healthy volunteers (Australian study; single ascending dose [SAD], *n* = 28, doses 1–25 mg/kg), in which no serious adverse events (SAEs) were observed (unpublished data).

Full analysis set (FAS) was defined as all patients who were successfully randomized and received at least one dose of ALMB-0166. The FAS was used for the analysis of demographic and baseline characteristics. Pharmacokinetic concentration analysis set (PKCS) included all patients who received ALMB-0166 and had at least one measurable concentration. Pharmacokinetic parameter analysis set (PKPS) included all patients who received ALMB-0166 and had at least one evaluable PK parameter. Safety analysis set (SS) included all patients who received at least one dose of ALMB-0166. Immunogenicity analysis set (IS) included all enrolled patients who had received at least one dose of ALMB-0166 and had baseline and at least one post-baseline immunogenicity evaluation data. Pharmacodynamic analysis set (PDS) consisted of all patients enrolled who had received at least one dose of ALMB-0166 with data from baseline and at least one post-baseline pharmacodynamic evaluation.

Safety analyses were performed using descriptive statistics. The efficacy analysis through Day 56 was based on the FAS and summarized descriptively. The ISNCSCI sensory scores, motor scores, injury grades and VAS scores were summarized by dose group, along with changes from baseline for each indicator. To preliminarily explore treatment differences, a *post hoc* comparison between ALMB-0166 and placebo was conducted for efficacy outcomes through Day 28 using a mixed-model for repeated measures (MMRM). The MMRM included baseline score as a covariate, with visit, treatment group and visit-by-treatment interaction as fixed effects, and was tested at a two-sided *α* level of 0.05. Statistical analyses were performed using SAS version 9.4. The PK parameters of ALMB-0166 were calculated using non-compartmental methods with Phoenix WinNonlin version 8.3.5. A power function model was used to evaluate the linear relationship between exposure (*C*_max_, AUC_0-t_ and AUC_0-∞_) and dose by the following equation: ln(*y*) = *β*_0_ + *β*_1_ × ln(Dose). A dose-proportional increase was indicated if the 90% confidence interval (CI) for the slope *β*_1_ included 1.

For categorical variables, data were summarized as frequency counts and corresponding percentages. For continuous variables, results were presented as the number of patients (*n*), mean and standard deviation, median, minimum and maximum. For variables potentially deviating from normality, the number of patients, median, interquartile range, minimum and maximum are reported. When applicable, the geometric mean and its coefficient of variation were provided.

## Results

### Patients

**Figure 1:**
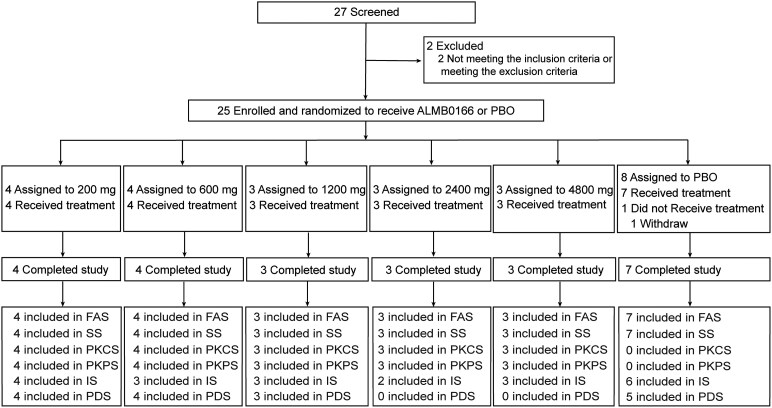
**Study flowchart.** PBO, placebo; FAS, full analysis set; SS, safety set; PKCS, pharmacokinetic concentration analysis set; PKPS, pharmacokinetic parameter analysis set; IS, immunogenicity analysis set; PDS, pharmacodynamic analysis set.

Between 1 July 2022 and 17 October 2024, a total of 25 patients were enrolled across 7 study sites across China. One patient (4.0%) withdrew after randomization and 24 patients (96.0%) received treatment: four in each of the 200 and 600 mg groups, three in each of the 1200, 2400 and 4800 mg groups and seven in the placebo group. All 24 patients who received study treatment completed the trial. The study flowchart is shown in Fig. 1. Twenty-four patients were included in the FAS and SS, 17 patients in the PKCS and PKPS, 21 patients in the IS and 16 patients in the PDS. The median age of patients was 57.5 years (range: 39–74), 70.8% were male, and the mean (standard deviation, SD) weight was 72.2 (13.6) kg. Most patients had AIS Grade C (20 patients, 83.3%). The number and percentage of patients with spinal injury sites involving C3, C4, C5, C6 and C7 were 6 (25.0%), 16 (66.7%), 18 (75.0%), 14 (58.3%) and 4 (16.7%), respectively. Overall, 66.7% (16/24) of patients received concomitant analgesics (58.8% [10/17] in the ALMB-0166 all dose groups versus 86.7% [6/7] in the placebo group). Demographic and baseline characteristics of all enrolled patients are displayed in Table 1.

**Table 1:** Patient demographics and baseline characteristics (FAS)

Characteristics	All (*n* = 24)
Age, years, median (range)	57.5 (39, 74)
Sex	
Female	7 (29.2)
Male	17 (70.8)
Height, cm, mean (SD)	168.6 (9.0)
Weight, kg, mean (SD)	72.2 (13.6)
AIS grade	
B	4 (16.7)
C	20 (83.3)
Neurological level of injury	
C3	6 (25.0)
C4	11 (45.8)
C5	6 (25.0)
C6	1 (4.2)
Site of spinal injury^a^	
C3	6 (25.0)
C4	16 (66.7)
C5	18 (75.0)
C6	14 (58.3)
C7	4 (16.7)

Abbreviations: AIS, American Spinal Injury Association Impairment Scale; SD, standard deviation.

*Note*: Data are presented as no. (%) unless otherwise noted. *n*: number of patients.

^a^A patient may have more than one site of spinal injury.

### Safety

Based on SS, treatment-emergent adverse events (TEAEs) were reported in 94.1% (16/17) of patients in the ALMB-0166 all dose groups and 100% (7/7) in the placebo group. Treatment-related adverse events (TRAEs) occurred in 35.3% (6/17) and 28.6% (2/7) of patients in the ALMB-0166 all dose and placebo groups, respectively. The most common TEAEs (incidence ≥20%) in the ALMB-0166 all dose groups were hyponatremia (35.3%, 6/17), followed by hypokalaemia, constipation, pyrexia and anaemia (29.4% each, 5/17). Grade ≥3 TEAEs were observed in 17.6% (3/17) of patients in the ALMB-0166 all dose groups, including one case each (5.9%, 1/17) of hypokalaemia, pneumonia, respiratory failure and deep vein thrombosis. In the placebo group, the most common TEAEs were hyponatremia, constipation, sinus bradycardia and urinary tract infection (42.9% each, 3/7), followed by hypokalaemia and pyrexia (28.6% each, 2/7). In the placebo group, 42.9% (3/7) of patients experienced Grade ≥3 TEAEs, including hypokalaemia in two patients (28.6%, 2/7) and hyponatremia in one (14.3%, 1/7). In the ALMB-0166 all dose groups, TRAEs included one case each (1/17, 5.9%) of sinus bradycardia, atrial fibrillation, elevated alanine aminotransferase, positive urinary erythrocytes, positive urinary occult blood, detectable urinary glucose, elevated aspartate aminotransferase, elevated percentage of reticulocytes, elevated reticulocyte count and infusion-related reactions (a broad term encompassing intravenous administration-associated adverse reactions, including pyrogen reactions, allergic reactions, pyrogen-like reactions, bacterial contamination reactions); all of these events were Grade 1. In the placebo group, TRAEs occurred in two patients (28.6%, 2/7), both presenting as Grade 1 sinus bradycardia. One SAE (5.9%, 1/17) of deep vein thrombosis was reported in the ALMB-0166 all dose groups and was assessed as possibly unrelated to the study drug; no SAEs were reported in the placebo group. No patients in either group experienced a TEAE leading to death. All TEAEs and TRAEs are summarized by group in Supplementary Tables 1 and 2, respectively. All Grade 3 or higher TEAEs and all SAEs are presented as individual case listings in Supplementary Table 3.

### PK and immunogenicity analysis

**Figure 2:**
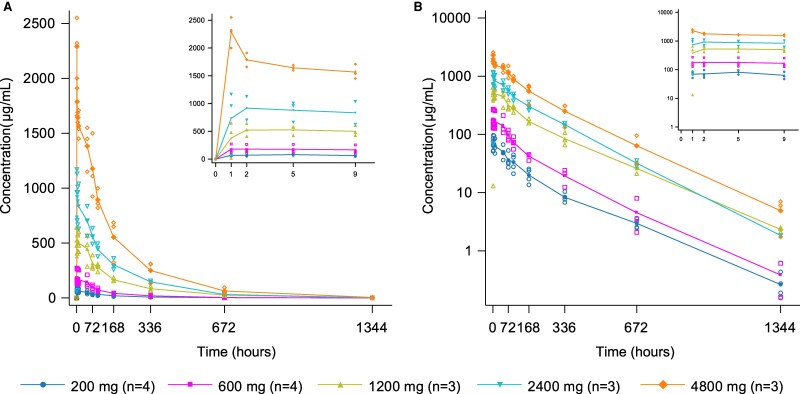
**Pharmacokinetics profiles of 0166.** (**A**) Linear plot of the mean concentration–time curve; (**B**) Semilogarithmic plot of the mean concentration–time curve. Pharmacokinetic parameters across all groups through Day 56 were summarized using descriptive statistics. Each symbol represents an individual patient. Hollow symbols represent individual patient values at each time point. Solid symbol indicates the estimated mean.

After administration of ALMB-0166 at doses of 200, 600, 1200, 2400 or 4800 mg, the time course of serum concentrations was similar across dose groups: concentrations rose gradually from the start of the infusion, peaked essentially at the end of infusion and then declined slowly. The mean serum concentration-time curves for each group are shown in Fig. 2. The median *T*_max_ of ALMB-0166 in serum ranged from 1.03 to 2.01 h across dose groups. Systemic exposure to ALMB-0166 increased with increasing dose. Mean *C*_max_ values ranged from 76.80 to 2290.00 μg/ml, and mean AUC_0-t_ and AUC_0-∞_ ranged from 9.80 to 256.06 h × mg/ml and from 9.88 to 257.29 h × mg/ml, respectively. The terminal half-life, volume of distribution and clearance of ALMB-0166 did not differ significantly among dose groups, with ranges of 149.86–201.24 h, 4.00–6.34 l and 14.29–30.94 ml/h, respectively. A summary of the PK parameters of ALMB-0166 is shown in Table 2. In the dose range of 200–4800 mg, the *β*-values for *C*_max_, AUC_0-t_ and AUC_0-∞_ were 1.122 (95% CI 1.020–1.224), 1.088 (0.929–1.246) and 1.084 (0.956–1.212), respectively. All *β*-values were close to 1, and the 95% CI either included or approximated 1, indicating that ALMB-0166 exhibited essentially linear PK over the 200–4800 mg dose range.

**Table 2:** Pharmacokinetic parameters of ALMB-0166

Parameter	200 mg	600 mg	1200 mg	2400 mg	4800 mg
*T*_max_ (h)	1.60 (1.03–4.98)	2.01 (0.98–2.07)	1.03 (1.02–9.02)	1.08 (1.02–1.95)	1.07 (1.03–1.10)
*C*_max_ (µg/ml)	76.80 (18.2)	181.75 (34.2)	575.67 (15.2)	946.33 (24.6)	2290.00 (12.1)
AUC_0-t_ (h × mg/ml)	9.80 (27.3)	19.57 (41.9)	84.44 (13.6)	108.57 (30.2)	256.06 (21.2)
AUC_0-∞_ (h × mg/ml)	9.88 (27.4)	21.01 (32.0)	85.09 (13.6)	126.81 (15.1)	257.29 (21.3)
*t*_1/2_ (h)	201.24 (3.5)	151.66 (37.4)	193.27 (4.6)	149.86 (36.2)	175.92 (8.1)
*V* (L)	6.22 (28.2)	6.34 (34.3)	4.00 (19.1)	4.10 (35.7)	4.84 (16.2)
CL (ml/h)	21.38 (26.5)	30.94 (32.3)	14.29 (14.6)	19.20 (14.0)	19.32 (24.3)

Data were described by mean (CV%), except for *T*_max_ of median (range). AUC_0-t_: area under the concentration–time curve from time zero to time of the last measurable concentration; AUC_0-∞_: area under the concentration–time curve from time zero to infinity; CL: clearance; *C*_max_: maximum concentration; CV, coefficient of variation; *T*_max_: time to maximum concentration; *t*_1/2_: terminal half-life; *V*: volume of distribution.

Based on IS, one patient each in the 200 mg group, 600 mg group and placebo group who were negative for ADA at baseline developed at least one ADA-positive sample post-baseline. The low incidence of ADA positivity, which was not dose-related, together with the low titres of ADA-positive samples, suggested a low risk of immunogenicity for ALMB-0166.

### Efficacy

#### ISNCSCI sensory scores

**Figure 3:**
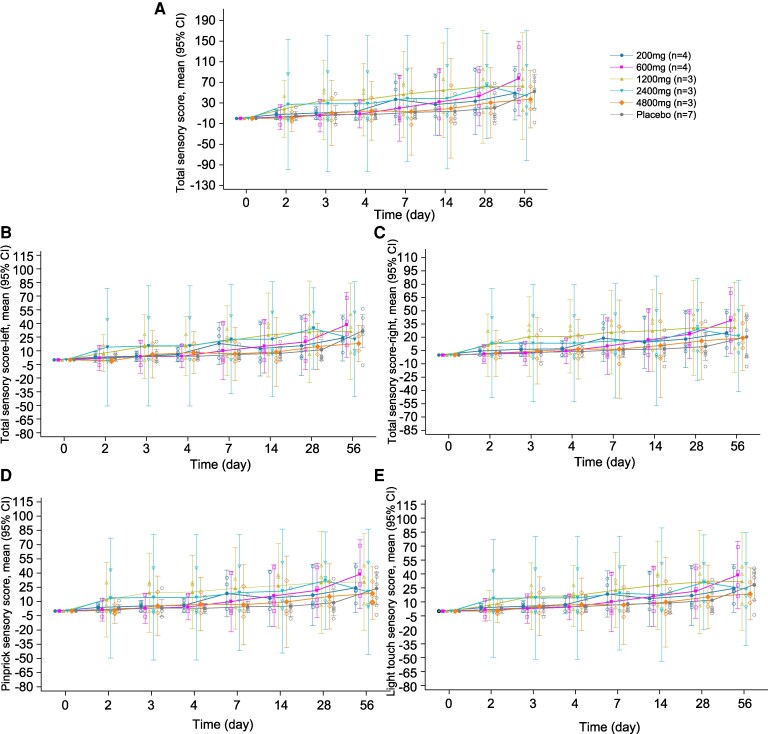
**Changes from baseline in ISNCSCI sensory scores.** (**A**) Total sensory score; (**B**) total sensory score-left side; (**C**) total sensory score-right side; (**D**) pinprick sensory score; (**E**) light touch sensory score. Changes from baseline in ISNCSCI sensory scores across all groups through Day 56 were summarized using descriptive statistics. Hollow symbols represent individual patient values at each time point. Solid symbol indicates the estimated mean. Data are presented as mean (95% CI). ISNCSCI, International Standards for Neurological Classification of Spinal Cord Injury.

**Figure 4:**
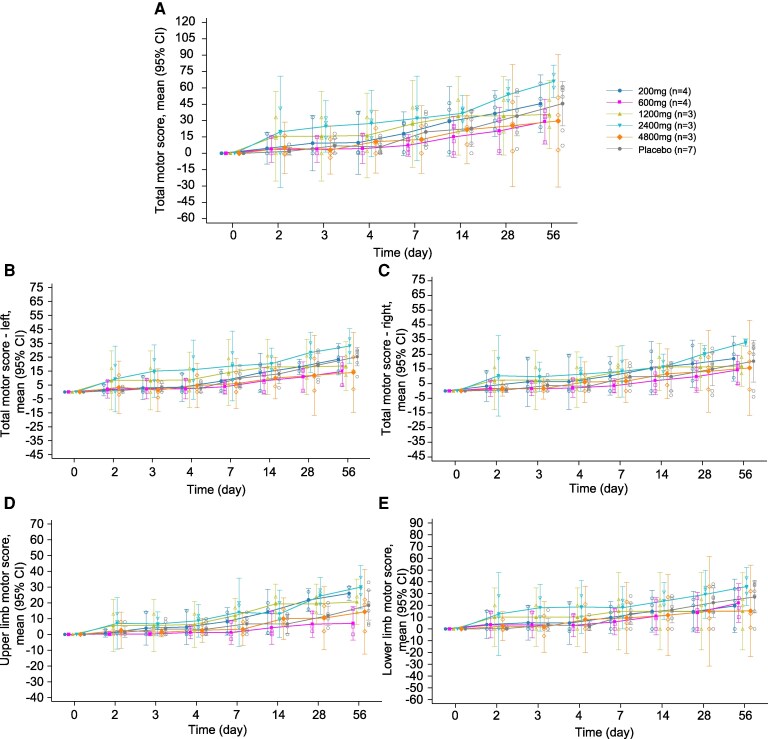
**Changes from baseline in ISNCSCI sensory scores of pooled target dose groups versus placebo.** (**A**) Total sensory score; (**B**) total sensory score-left side; (**C**) total sensory score-right side; (**D**) pinprick sensory score; (**E**) light touch sensory score. The dose groups of 1200 and 2400 mg were pooled. Hollow symbols represent individual patient values at each time point. Solid symbol indicates the estimated mean. Data are presented as mean (95% CI). *P < 0.05, **P < 0.01 versus placebo. A *post hoc* comparison between the pooled ALMB-0166 groups (1200 and 2400 mg) and placebo was performed for efficacy outcomes through Day 28 using a mixed-model for repeated measures (MMRM). The model included baseline score as a covariate, with visit, treatment group and visit-by-treatment interaction as fixed effects, and was tested at a two-sided *α* level of 0.05. ISNCSCI, International Standards for Neurological Classification of Spinal Cord Injury.

The changes from baseline in ISNCSCI sensory scores by dose group over time are shown in Fig. 3. At Day 28, the changes from baseline in total sensory score, total sensory score-left, total sensory score-right, pinprick sensory score and light touch score were all numerically higher in all ALMB-0166 dose groups than in the placebo group (Fig. 3). Total sensory score improved from baseline in all groups. Except for the 4800 mg group, the improvement in total sensory score increased with increasing dose, with superior efficacy observed in the 1200 and 2400 mg groups. At Day 28, the mean change from baseline in total sensory score was 62.3 in the 1200 group, 63.0 in the 2400 mg group and 20.6 in the placebo group. Similar improvements were seen for total sensory score-left and total sensory score-right. At Day 28, the mean changes from baseline in pinprick sensory scores were 30.0 in the 1200, 32.0 in the 2400 mg group and 8.7 in the placebo group. For light touch sensory score, the mean changes from baseline were 31.3 (1200 mg), 31.0 (2400 mg) and 11.9 (placebo). For exploratory purposes, patients from 1200 and 2400 mg dose groups were pooled. In this *post hoc* pooled analysis, the pooled active group showed numerically higher mean changes in sensory scores compared with the placebo group at all assessed time points, with nominal *P* < 0.05 (unadjusted for multiple comparisons) at most time points (Fig. 4). Additional *post hoc* exploratory comparisons of total sensory scores for each ALMB-0166 dose group versus placebo are presented in Supplementary Table 4.

#### ISNCSCI motor scores

**Figure 5:**
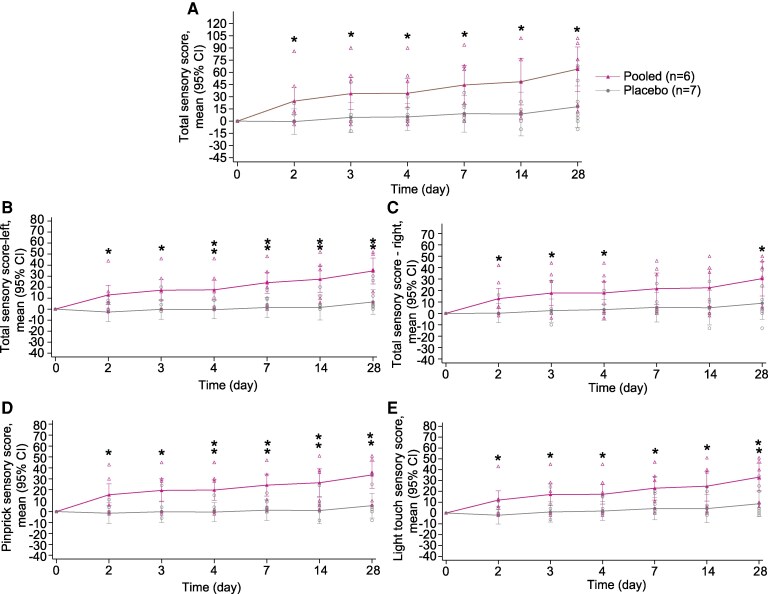
**Changes from baseline in ISNCSCI motor scores.** (**A**) Total motor score; (**B**) total motor score-left side; (**C**) total motor score-right side; (**D**) upper limb motor score and (**E**) lower limb motor score. Changes from baseline in ISNCSCI motor scores across all groups through Day 56 were summarized using descriptive statistics. Hollow symbols represent individual patient values at each time point. Solid symbol indicates the estimated mean. Data are presented as mean (95% CI). ISNCSCI, International Standards for Neurological Classification of Spinal Cord Injury.

**Figure 6:**
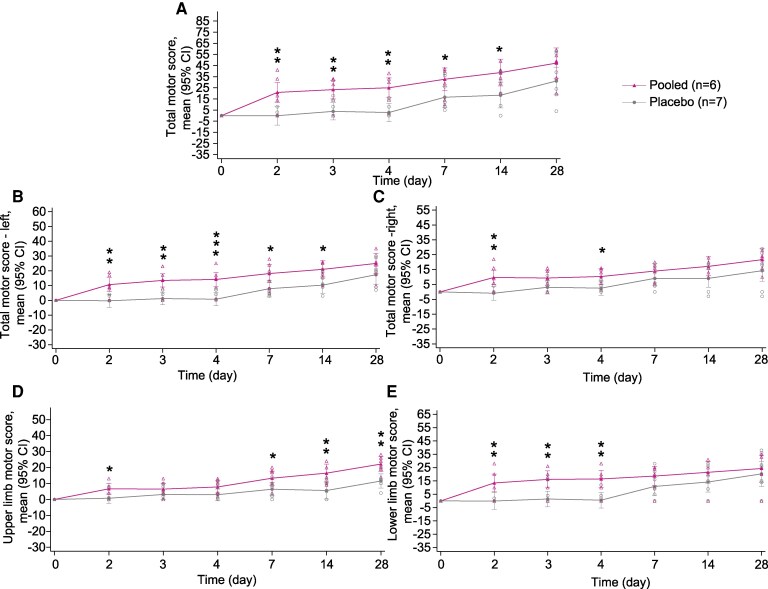
**Changes from baseline in ISNCSCI motor scores of pooled target dose groups versus placebo.** (**A**) Total motor score; (**B**) total motor score-left side; (**C**) total motor score-right side; (**D**) upper limb motor score; (**E**) lower limb motor score. The dose groups of 1200 and 2400 mg were pooled. Hollow symbols represent individual patient values at each time point. Solid symbol indicates the estimated mean. Data are presented as mean (95% CI). *P < 0.05, **P < 0.01, ***P < 0.001 versus placebo. A *post hoc* comparison between the pooled ALMB-0166 groups (1200 and 2400 mg) and placebo was performed for efficacy outcomes through Day 28 using a mixed-model for repeated measures (MMRM). The model included baseline score as a covariate, with visit, treatment group and visit-by-treatment interaction as fixed effects, and was tested at a two-sided *α* level of 0.05. ISNCSCI, International Standards for Neurological Classification of Spinal Cord Injury.

The changes from baseline in ISNCSCI motor scores by dose group over time are shown in Fig. 5. The mean change from baseline in motor scores showed a progressive upward trend in all groups over time. At Day 28, patients treated with ALMB-0166 exhibited improved motor function compared with those receiving placebo, with a mean increased from baseline of 53.7 in the 2400 mg group and 34.1 in the placebo group. Similar improvements were observed for total motor score—left and total motor score—right. At Day 28, the mean change from baseline in upper limb motor scores was higher in the 200, 1200 and 2400 mg groups than in the placebo group (21.8, 19.3, 24.3, respectively, versus 11.9 for placebo). Regarding the lower limb motor score at Day 28, patients in the 2400 mg group had a greater mean change from baseline values than those in the placebo group (29.3 versus 22.3). In the *post hoc* analysis, the pooled active group showed that ALMB-0166 had a rapid onset of effect, with numerically greater mean improvements from baseline in motor scores compared with the placebo group at all early time points; nominal *P*-values were <0.05 (unadjusted for multiple comparisons) was observed at these time points (Fig. 6). Additional *post hoc* exploratory comparisons of total motor scores for each ALMB-0166 dose group versus placebo are presented in Supplementary Table 5.

#### ISNCSCI injury grades

Based on the FAS, a trend towards improvement in injury grade was observed in each group. The 1200 and 2400 mg dose groups showed a more favourable trend compared with the placebo group. Two patients in the ALMB-0166 group recovered from Grade C to Grade E, whereas no patient in the placebo group recovered to Grade E.

#### VAS scores

Patients in the 1200 and 2400 mg groups had a trend towards lower mean changes from baseline in VAS scores compared with the placebo group. At Day 28, VAS scores decreased by 40.0 in the 1200 mg group and 23.7 in the 2400 mg group compared with a decrease of 18.9 in the placebo group. At Day 56, VAS scores decreased by 44.3 in the 1200 mg group and 23.7 in the 2400 mg group, versus 20.9 in the placebo group (Supplementary Fig. 1).

## Discussion

This multicentre, randomized, double-blind, placebo-controlled, single-dose escalation Phase I/II study evaluated the safety, tolerability, PKs and effectiveness of ALMB-0166 in patients with acute SCI. The trial enrolled adult patients with SCI at or below the C3 segment and AIS grade of B or C. Preliminary results indicated that a single dose of ALMB-0166 was generally well tolerated and led to greater improvement in neurological function compared with placebo in this population.

ALMB-0166 is a monoclonal antibody targeting human Cx43 hemichannels, with no approved analogous products currently available domestically or internationally. Preclinical studies, together with an initial Phase I study in healthy volunteers in Australia, have demonstrated the favourable safety and tolerability of ALMB-0166 (unpublished data). In preclinical multiple-dosing trials on mice and cynomolgus monkeys, doses up to 250 mg/kg produced no drug-related systemic toxic responses or identifiable toxic target organs (unpublished data). Additionally, no drug-related serious local irritation reactions were observed at the injection site. In the first Australian Phase I study, 12 of 28 subjects (42.9%) who received ALMB-0166 experienced TEAEs. All TEAEs were mild to moderate in severity, and the majority were considered unrelated to ALMB-0166 (unpublished data). Clinical studies conducted to date indicate that although conventional candidate agents for acute SCI, such as methylprednisolone,^16^ riluzole^17^ and minocycline,^18^ may offer some neurological recovery benefits, they are associated with considerable safety concerns. These include systemic immunosuppression caused by high-dose glucocorticoids, riluzole-related hepatic burden and the complexity of regimen implementation in real-world practice. In this Phase I/II trial involving patients with SCI, ALMB-0166 also exhibited an acceptable preliminary safety profile. The incidence of TEAEs was 94.1% in the ALMB-0166 group (100% in the placebo group), with the majority (82.4%) being Grades 1 and 2. The incidence of TEAEs related to the study drug was 35.3% in the ALMB-0166 group (28.6% in the placebo group); all such events were Grade 1. There were no deaths or treatment-related SAEs. No new safety concerns were identified. Although patients with acute SCI are vulnerable to multiple medical complications,^2^ the type and severity of AEs and SAEs observed in this study confirmed an acceptable safety profile for ALMB-0166.

Following a single intravenous infusion of ALMB-0166 at doses ranging from 200 to 4800 mg, systemic exposure increased with the administered dose in an essentially linear PK profile. This finding was similar to previous PK results observed in healthy volunteers in the Phase I study. The PK profile of ALMB-0166 was also consistent with the known PK characteristics of monoclonal antibodies.^19^ Regarding immunogenicity, one patient in the placebo group tested positive for ADA. In the ALMB-0166 groups, one patient each in the 200 and 600 mg groups who were ADA-negative at baseline became ADA-positive post-baseline. The lower incidence of ADA positivity, which did not show a significant dose-related trend, together with the lower titre of ADA-positive samples, suggested a low risk of immunogenicity for ALMB-0166. This is consistent with the immunogenicity results from the Australian Phase I study and with previous reports that therapeutic targeting of Cx43 was not associated with immunological responses or local or systemic AEs.^20^

ALMB-0166 prevents the opening of Cx43 hemichannels induced by cell membrane damage, inhibits the release of pro-inflammatory factors from astrocytes and limits the spread of secondary injury. In patients with acute SCI, several dose groups of ALMB-0166 exhibited greater change from baseline than the placebo group across multiple ISNCSCI sensory measures, including ISNCSCI total sensory scores, total sensory scores—left, total sensory scores—right, pinprick sensory score and light touch sensory score. Notably, the 1200 and 2400 mg dose groups achieved superior results on these sensory endpoints. A similar trend was observed for motor scores: total motor score, total motor score-left, total motor score-right, upper limb motor score and lower limb motor score all showed greater change from baseline than those in the placebo group. In addition, pooled analysis of the 1200 and 2400 mg groups demonstrated better improvements in both sensory scores and motor scores than those in the placebo group. In terms of injury grading, the 1200 and 2400 mg groups also showed a more pronounced trend towards improvement relative to placebo. Collectively, these findings indicated that ALMB-0166 has beneficial effects on of sensory-motor recovery in acute SCI. Furthermore, with respect to pain relief, the 1200 and 2400 mg groups also showed a trend towards greater pain relief than the placebo group. Based on the comprehensive safety and efficacy results, the target doses for further studies were determined as 1200 and 2400 mg.

In the present study, a relative decline in response was observed in the 4800 mg group, despite essentially linear PKs across the 200–4800 mg dose range and the absence of SAEs at the highest dose. Potential mechanisms include over-suppression of hemichannels, which may disrupt astrocytic homeostasis. While moderate inhibition is therapeutic, very high exposures may impair gap junction communication essential for astrocytic and neuronal support.^21,22^ Additionally, pharmacodynamics may saturate at supra-therapeutic levels, and excessive blockade could trigger compensatory inflammatory pathways, reducing net benefit without overt toxicity.^22^ It is important to note that these mechanistic explanations remain hypothetical, and further investigation is required to elucidate the precise action of ALMB-0166 in SCI.

This study has several limitations. First, it employed a SAD design. However, for early-phase trials in vulnerable SCI populations, this approach was chosen to prioritize initial safety, tolerability and PK characterization while minimizing cumulative exposure risks before a safe dose range is established. Second, the follow-up duration was limited to 56 days, which was insufficient to assess long-term AEs and durable effects. Nevertheless, this period encompasses multiple drug half-lives, allowing for near-complete elimination. Third, the sample sizes for each dose group were small, limiting the statistical power to detect significant effects; therefore, only preliminary efficacy results could be provided. Fourth, the 2000 ISNCSCI revision was used for neurological assessments, rather than the current 2019 standard. As the earlier version is still widely adopted and well standardized in China, and because efficacy outcomes in this early-phase trial were exploratory, its use is not considered to compromise the reliability of the main conclusions. Fifth, the potential confounding influence of concomitant analgesic use was not adjusted for in the analysis of VAS scores. Together, these limitations highlight the need for larger, longer-term trials—including multiple ascending dose studies with assessments based on the 2019 ISNCSCI standards—to determine optimal dosing and validate the therapeutic potential of ALMB-0166 in acute SCI.

## Conclusion

ALMB-0166 demonstrated a manageable safety profile and improved neurologic recovery in patients with acute SCI. Based on the integrated results of safety, efficacy and PK, 1200 and 2400 mg were recommended as the therapeutic doses for further exploration in the subsequent clinical trial of ALMB-0166.

## Supplementary Material

fcag275_Supplementary_Data

## Data Availability

The datasets generated during and/or analysed during the current study are available from the corresponding author upon reasonable request. The code for this analysis is available on GitHub at https://github.com/lcy2010/SAS-code.git.
