# Supplementary material for: Safety, tolerability and preliminary efficacy of ALMB-0166 in patients with acute spine cord injury
Source: Brain Commun. 2026 Jul 25;8(4):fcag275. doi: 10.1093/braincomms/fcag275 (PMC13421776; doi:10.1093/braincomms/fcag275)
Supplement: fcag275_Supplementary_Data [file fcag275_supplementary_data.docx]

**Supplementary material**

**Supplementary Figure 1.** Change from baseline in VAS scores in 1200 mg, 2400 mg and Placebo groups.

**Supplementary Table 1:** All TEAEs across All Groups

**Supplementary Table 2.** All TRAEs across All Groups

**Supplementary Table 3.** Casuistic overview of Grade ≥ 3 TEAEs and SAEs

**Supplementary Table 4.** Changes from baseline in ISNCSCI total sensory scores

**Supplementary Table 5.** Changes from baseline in ISNCSCI total motor scores


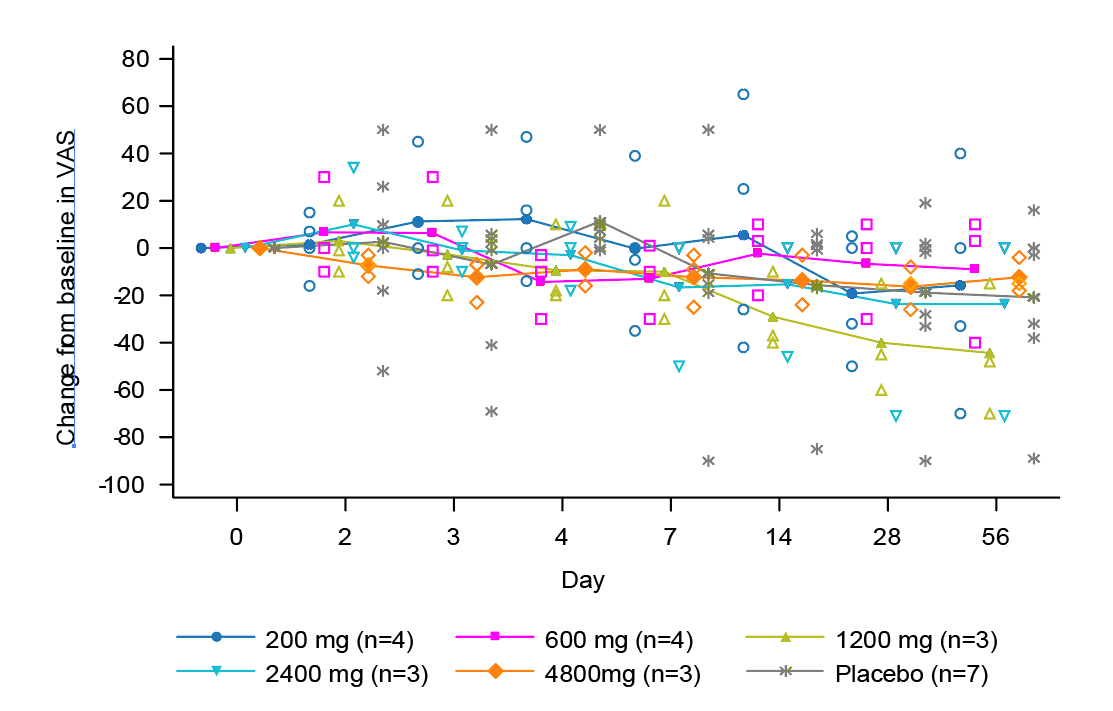


**Supplementary Figure 1.** Change from baseline in VAS scores in 1200 mg, 2400 mg and Placebo groups. Changes from baseline in VAS scores across groups through Day 56 were summarized using descriptive statistics. Hollow symbols represent individual patient values at each time point. Solid symbol indicates the estimated mean. For groups with N < 10, all individual data points are shown. VAS, Visual Analogue Scale.

**Supplementary Table 1. All TEAEs across All Groups**

|  | 200mg N=4 | 600mg N=4 | 1200mg N=3 | 2400mg N=3 | 4800mg N=3 | ALMB-0166  All doses N=17 | PBO N=7 | ALL N=24 |
| --- | --- | --- | --- | --- | --- | --- | --- | --- |
| TEAEs | 4 (100) | 4 (100) | 3 (100) | 2 (66.7) | 3 (100) | 16 (94.1) | 7 (100) | 23 (95.8) |
| Grade≥3 TEAEs | 0 | 2 (50.0) | 0 | 0 | 1 (33.3) | 3 (17.6) | 3 (42.9) | 6 (25.0) |
| SAEs | 0 | 1 (25.0) | 0 | 0 | 0 | 1 (5.9) | 0 | 1 (4.2) |
| TEAEs leading to death | 0 | 0 | 0 | 0 | 0 | 0 | 0 | 0 |
| TEAEs leading to discontinue treatment | 0 | 0 | 0 | 0 | 0 | 0 | 0 | 0 |
| All TEAEs | | | | | | | | |
| Hyponatremia | 3 (75.0) | 2 (50.0) | 0 | 1 (33.3) | 0 | 6 (35.3) | 3 (42.9) | 9 (37.5) |
| Hypokalemia | 3 (75.0) | 2 (50.0) | 0 | 0 | 0 | 5 (29.4) | 2 (28.6) | 7 (29.2) |
| Hypoalbuminemia | 0 | 1 (25.0) | 0 | 1 (33.3) | 0 | 2 (11.8) | 0 | 2 (8.3) |
| Hyperkalemia | 1 (25.0) | 0 | 0 | 0 | 0 | 1 (5.9) | 1 (14.3) | 2 (8.3) |
| Hypoproteinemia | 0 | 0 | 0 | 1 (33.3) | 0 | 1 (5.9) | 0 | 1 (4.2) |
| Hypocalcemia | 0 | 0 | 0 | 1 (33.3) | 0 | 1 (5.9) | 0 | 1 (4.2) |
| Hypochloremia | 0 | 1 (25.0) | 0 | 0 | 0 | 1 (5.9) | 0 | 1 (4.2) |
| Hyperuricemia | 0 | 0 | 0 | 0 | 0 | 0 | 1 (14.3) | 1 (4.2) |
| IGT | 0 | 0 | 0 | 1 (33.3) | 0 | 1 (5.9) | 0 | 1 (4.2) |
| Diabetes | 0 | 0 | 0 | 1 (33.3) | 0 | 1 (5.9) | 0 | 1 (4.2) |
| Leukocytosis | 1 (25.0) | 1 (25.0) | 0 | 1 (33.3) | 0 | 3 (17.6) | 1 (14.3) | 4 (16.7) |
| Elevated ALT | 0 | 2 (50.0) | 0 | 1 (33.3) | 0 | 3 (17.6) | 1 (14.3) | 4 (16.7) |
| Elevated AST | 0 | 2 (50.0) | 0 | 1 (33.3) | 0 | 3 (17.6) | 1 (14.3) | 4 (16.7) |
| Elevated CRP | 1 (25.0) | 1 (25.0) | 0 | 0 | 0 | 2 (11.8) | 1 (14.3) | 3 (12.5) |
| Positive urine RBCs | 1 (25.0) | 1 (25.0) | 0 | 1 (33.3) | 0 | 3 (17.6) | 0 | 3 (12.5) |
| Positive urine OB | 1 (25.0) | 1 (25.0) | 0 | 0 | 0 | 2 (11.8) | 1 (14.3) | 3 (12.5) |
| Elevated D-Dimer | 2 (50.0) | 1 (25.0) | 0 | 0 | 0 | 3 (17.6) | 0 | 3 (12.5) |
| Glycosuria | 1 (25.0) | 0 | 1 (33.3) | 0 | 0 | 2 (11.8) | 0 | 2 (8.3) |
| Elevated ESR | 1 (25.0) | 0 | 0 | 0 | 0 | 1 (5.9) | 0 | 1 (4.2) |
| Elevated reticulocyte percentage | 1 (25.0) | 0 | 0 | 0 | 0 | 1 (5.9) | 0 | 1 (4.2) |
| Elevated reticulocyte count | 1 (25.0) | 0 | 0 | 0 | 0 | 1 (5.9) | 0 | 1 (4.2) |
| Elevated serum CPK | 0 | 0 | 0 | 1 (33.3) | 0 | 1 (5.9) | 0 | 1 (4.2) |
| Elevated serum LDH | 0 | 0 | 0 | 1 (33.3) | 0 | 1 (5.9) | 0 | 1 (4.2) |
| Elevated serum FIB | 1 (25.0) | 0 | 0 | 0 | 0 | 1 (5.9) | 0 | 1 (4.2) |
| Thrombocytopenia | 0 | 1 (25.0) | 0 | 0 | 0 | 1 (5.9) | 0 | 1 (4.2) |
| Thrombocytopenia | 0 | 0 | 0 | 1 (33.3) | 0 | 1 (5.9) | 0 | 1 (4.2) |
| Constipation | 1 (25.0) | 1 (25.0) | 1 (33.3) | 0 | 2 (66.7) | 5 (29.4) | 3 (42.9) | 8 (33.3) |
| Diarrhea | 0 | 1 (25.0) | 0 | 0 | 1 (33.3) | 2 (11.8) | 1 (14.3) | 3 (12.5) |
| FGID | 0 | 0 | 0 | 0 | 0 | 0 | 1 (14.3) | 1 (4.2) |
| Neurogenic bowel | 0 | 1 (25.0) | 0 | 0 | 0 | 1 (5.9) | 0 | 1 (4.2) |
| Pyrexia | 1 (25.0) | 1 (25.0) | 1 (33.3) | 0 | 2 (66.7) | 5 (29.4) | 2 (28.6) | 7 (29.2) |
| Chest discomfort | 0 | 0 | 1 (33.3) | 0 | 0 | 1 (5.9) | 0 | 1 (4.2) |
| Sinus bradycardia | 0 | 1 (25.0) | 0 | 0 | 1 (33.3) | 2 (11.8) | 3 (42.9) | 5 (20.8) |
| Atrial fibrillation | 0 | 0 | 0 | 1 (33.3) | 0 | 1 (5.9) | 0 | 1 (4.2) |
| Arrhythmia | 0 | 1 (25.0) | 0 | 0 | 0 | 1 (5.9) | 0 | 1 (4.2) |
| RBBB | 0 | 1 (25.0) | 0 | 0 | 0 | 1 (5.9) | 0 | 1 (4.2) |
| Anemia | 1 (25.0) | 3 (75.0) | 0 | 1 (33.3) | 0 | 5 (29.4) | 1 (14.3) | 6 (25.0) |
| Urinary tract infection | 0 | 1 (25.0) | 0 | 1 (33.3) | 0 | 2 (11.8) | 3 (42.9) | 5 (20.8) |
| COVID-19 | 0 | 1 (25.0) | 0 | 0 | 0 | 1 (5.9) | 0 | 1 (4.2) |
| S. wound infection | 0 | 1 (25.0) | 0 | 0 | 0 | 1 (5.9) | 0 | 1 (4.2) |
| URTI | 0 | 0 | 0 | 1 (33.3) | 0 | 1 (5.9) | 0 | 1 (4.2) |
| **Procedural pain** | 0 | 0 | 1 (33.3) | 1 (33.3) | 0 | 2 (11.8) | 0 | 2 (8.3) |
| Cervical spine fracture | 0 | 0 | 0 | 0 | 0 | 0 | 1 (14.3) | 1 (4.2) |
| IRR | 1 (25.0) | 0 | 0 | 0 | 0 | 1 (5.9) | 0 | 1 (4.2) |
| Postoperative delirium | 0 | 0 | 0 | 0 | 0 | 0 | 1 (14.3) | 1 (4.2) |
| Pulmonary inflammation | 0 | 0 | 0 | 0 | 1 (33.3) | 1 (5.9) | 0 | 1 (4.2) |
| Respiratory failure | 0 | 0 | 0 | 0 | 1 (33.3) | 1 (5.9) | 0 | 1 (4.2) |
| Oropharyngeal pain | 0 | 0 | 1 (33.3) | 0 | 0 | 1 (5.9) | 0 | 1 (4.2) |
| Productive cough | 0 | 0 | 1 (33.3) | 0 | 0 | 1 (5.9) | 0 | 1 (4.2) |
| Insomnia | 1 (25.0) | 0 | 0 | 0 | 0 | 1 (5.9) | 1 (14.3) | 2 (8.3) |
| Delirium | 0 | 0 | 0 | 0 | 1 (33.3) | 1 (5.9) | 0 | 1 (4.2) |
| DVT | 0 | 1 (25.0) | 0 | 0 | 1 (33.3) | 2 (11.8) | 1 (14.3) | 3 (12.5) |
| Metabolic encephalopathy | 0 | 0 | 0 | 0 | 0 | 0 | 1 (14.3) | 1 (4.2) |
| Neck pain | 0 | 0 | 1 (33.3) | 0 | 0 | 1 (5.9) | 0 | 1 (4.2) |
| Rash | 0 | 0 | 0 | 0 | 0 | 0 | 1 (14.3) | 1 (4.2) |
| Neurogenic bladder | 0 | 1 (25.0) | 0 | 0 | 0 | 1 (5.9) | 0 | 1 (4.2) |

TEAE, Treatment-Emergent Adverse Event. SAE, Serious Adverse Event. IGT, Impaired Glucose Tolerance. ALT, Alanine Aminotransferase. AST, Aspartate Aminotransferase. CRP, C-reactive Protein. RBC, Red Blood Cell. OB, occult blood. CPK, Creatine phosphokinase FIB, fibrinogen. LDH, lactate dehydrogenase. RSR, erythrocyte sedimentation rate. FGID, Functional gastrointestinal disorder. S. Staphylococcal. IRR, Infusion-related reaction. DVT, Deep vein thrombosis. URTI, Upper respiratory tract infection. RBBB, Right bundle branch block.

**Supplementary Table 2. All TRAEs across All Groups**

|  | 200mg N=4 | 600mg N=4 | 1200mg N=3 | 2400mg N=3 | 4800mg N=3 | ALMB-0166  All doses N=17 | PBO N=7 | All  N=24 |
| --- | --- | --- | --- | --- | --- | --- | --- | --- |
| **TRAEs** | 2 (50.0) | 0 | 1 (33.3) | 2 (66.7) | 1 (33.3) | 6 (35.3) | 2 (28.6) | 8 (33.3) |
| Grade≥3 TRAEs | 0 | 0 | 0 | 0 | 0 | 0 | 0 | 0 |
| Serious TRAEs | 0 | 0 | 0 | 0 | 0 | 0 | 0 | 0 |
| TRAEs leading to death | 0 | 0 | 0 | 0 | 0 | 0 | 0 | 0 |
| TRAEs leading to discontinue treatment | 0 | 0 | 0 | 0 | 0 | 0 | 0 | 0 |
| **All TRAEs** | | | | | | | | |
| Sinus bradycardia | 0 | 0 | 0 | 0 | 1 (33.3) | 1 (5.9) | 2 (28.6) | 3 (12.5) |
| Atrial fibrillation | 0 | 0 | 0 | 1 (33.3) | 0 | 1 (5.9) | 0 | 1 (4.2) |
| Elevated ALT | 0 | 0 | 0 | 1 (33.3) | 0 | 1 (5.9) | 0 | 1 (4.2) |
| Positive urine RBCs | 1 (25.0) | 0 | 0 | 0 | 0 | 1 (5.9) | 0 | 1 (4.2) |
| Positive urine OB | 1 (25.0) | 0 | 0 | 0 | 0 | 1 (5.9) | 0 | 1 (4.2) |
| Glycosuria | 0 | 0 | 1 (33.3) | 0 | 0 | 1 (5.9) | 0 | 1 (4.2) |
| Elevated AST | 0 | 0 | 0 | 1 (33.3) | 0 | 1 (5.9) | 0 | 1 (4.2) |
| Elevated reticulocyte percentage | 1 (25.0) | 0 | 0 | 0 | 0 | 1 (5.9) | 0 | 1 (4.2) |
| Elevated reticulocyte count | 1 (25.0) | 0 | 0 | 0 | 0 | 1 (5.9) | 0 | 1 (4.2) |
| IRR | 1 (25.0) | 0 | 0 | 0 | 0 | 1 (5.9) | 0 | 1 (4.2) |

TRAE, Treatment-Related Adverse Event. ALT, Alanine Aminotransferase. AST, Aspartate Aminotransferase. RBC, Red Blood Cell. OB, occult blood. IRR, Infusion-related reaction.

**Supplementary Table 3. Casuistic overview of Grade ≥ 3 TEAEs and SAEs**

|  | **TEAE** | **Grade** | **Date**  **Start/ End** | **Interventions to AEs** | **Relationship to ALMB-0166** | **Outcome** | **SAE or Not** |
| --- | --- | --- | --- | --- | --- | --- | --- |
| 600mg | Hypokalemia | 3 | 2023-08-18 | No treatment | Possibly unrelated | Unrecovered | No |
| 600mg | Deep Vein Thrombosis | 3 | 2023-04-25/  2023-05-09 | Medication/ Hospitalization | Possibly unrelated | Improved/  Resolved | Yes |
| 4800mg | Respiratory failure^*^ | 4 | 2024-07-12/  2024-07-17 | Non-pharmacological treatment/  Medication | Possibly unrelated | Recovered | No |
|  | Pulmonary inflammation^*^ | 3 | 2024-07-12/  2024-09-03 | Medication | Possibly unrelated | Recovered | No |
| PBO | Hypokalemia | 3 | 2022-10-21/  2022-10-22 | Medication | Possibly unrelated | Recovered | No |
| PBO | Hypokalemia | 3 | 2024-04-26/  2024-04-27 | Medication | Possibly unrelated | Recovered | No |
| PBO | Hyponatremia | 3 | 2024-06-09/  2024-06-13 | Medication | Unrelated | Recovered | No |

TEAE, Treatment-Emergent Adverse Event. SAE, Serious Adverse Event. PBO, Placebo. ^*^ Two AEs occurred in the same patient.

**Supplementary Table 4. Changes from baseline in ISNCSCI total sensory scores**

|  |  | 200mg N=4 | 600mg N=4 | 1200mg N=3 | 2400mg  N=3 | 4800mg N=3 | PBO N=7 |
| --- | --- | --- | --- | --- | --- | --- | --- |
| D2 | EM (SE) | 9.43 (9.95) | 1.46 (9.89) | 20.32 (11.45) | 28.04 (11.31) | 2.12 (11.34) | -0.44 (7.69) |
|  | EMD (SE)  vs. PBO | 9.87 (12.82) | 1.89 (12.33) | 20.75 (14.02) | 28.48 (13.76) | 2.56 (13.83) |  |
|  | 95% CI | (-16.76, 36.50) | (-23.75, 27.54) | (-8.37, 49.88) | (-0.13, 57.08) | (-26.20, 31.31) |  |
|  | P | 0.45 | 0.88 | 0.15 | 0.05 | 0.86 |  |
| D3 | EM (SE) | 12.43 (12.17) | 3.96 (12.12) | 37.32 (14.02) | 29.37 (13.91) | 11.78 (13.93) | 5.40 (9.30) |
|  | EMD (SE)  vs. PBO | 7.04 (15.56) | -1.44 (15.09) | 31.92 (17.05) | 23.97 (16.81) | 6.39 (16.88) |  |
|  | 95% CI | (-25.55, 39.62) | (-33.14, 30.26) | (-3.80, 67.64) | (-11.30, 59.25) | (-29.01, 41.79) |  |
|  | P | 0.66 | 0.93 | 0.077 | 0.17 | 0.71 |  |
| D4 | EM (SE) | 14.93 (10.98) | 6.96 (10.92) | 38.32 (12.64) | 29.37 (12.51) | 15.78 (12.54) | 5.97 (8.41) |
|  | EMD (SE)  vs. PBO | 8.96 (14.10) | 0.99 (13.57) | 32.35 (15.43) | 23.40 (15.17) | 9.82 (15.24) |  |
|  | 95% CI | (-20.81, 38.74) | (-27.78, 29.76) | (-0.26, 64.96) | (-8.70, 55.50) | (-22.43, 42.06) |  |
|  | P | 0.53 | 0.94 | 0.05 | 0.14 | 0.53 |  |
| D7 | EM (SE) | 38.93 (14.95) | 18.46 (14.91) | 48.65 (17.23) | 39.37 (17.14) | 14.45 (17.16) | 9.97 (11.38) |
|  | EMD (SE)  vs. PBO | 28.96 (18.98) | 8.49 (18.60) | 38.68 (20.83) | 29.40 (20.64) | 4.48 (20.70) |  |
|  | 95% CI | (-11.46, 69.40) | (-31.23, 48.21) | (-5.70, 83.07) | (-14.63, 73.44) | (-39.65, 48.62) |  |
|  | P | 0.15 | 0.65 | 0.08 | 0.17 | 0.83 |  |
| D14 | EM (SE) | 28.93 (17.48) | 30.46 (17.45) | 56.65 (20.17) | 39.37 (20.09) | 20.45 (20.10) | 9.73 (13.41) |
|  | EMD (SE)  vs. PBO | 19.20 (22.19) | 20.73 (21.88) | 46.92 (24.37) | 29.64 (24.20) | 10.72 (24.25) |  |
|  | 95% CI | (-27.45, 65.85) | (-25.36, 66.81) | (-4.32, 98.16) | (-21.30, 80.58) | (-40.30, 61.75) |  |
|  | P | 0.40 | 0.36 | 0.07 | 0.24 | 0.66 |  |
| D28 | EM (SE) | 35.43 (16.70) | 41.71 (16.67) | 63.32 (19.26) | 63.70 (19.18) | 31.78 (19.20) | 18.54 (12.70) |
|  | EMD (SE)  vs. PBO | 16.89 (21.16) | 23.17 (20.81) | 44.78 (23.24) | 45.17 (23.06) | 13.25 (23.11) |  |
|  | 95% CI | (-27.55, 61.33) | (-20.63, 66.96) | (-4.04, 93.60) | (-3.33, 93.66) | (-35.34, 61.83) |  |
|  | P | 0.44 | 0.28 | 0.07 | 0.07 | 0.57 |  |

A post-hoc comparison between ALMB‑0166 and placebo was conducted for efficacy outcomes through Day 28 using a Mixed‑Model for Repeated Measures (MMRM). The MMRM included baseline score as a covariate, with visit, treatment group, and visit‑by‑treatment interaction as fixed effects, and was tested at a two‑sided α level of 0.05. D, Day. EM, Estimated mean. SE, Standard Error. EMD, Estimated mean difference. PBO, Placebo.

**Supplementary Table 5. Changes from baseline in ISNCSCI total motor scores**

|  |  | 200mg N=4 | 600mg N=4 | 1200mg N=3 | 2400mg N=3 | 4800mg N=3 | PBO N=7 |
| --- | --- | --- | --- | --- | --- | --- | --- |
| D2 | EM (SE) | 8.66 (5.34) | 2.61 (5.03) | 22.94 (6.62) | 18.50 (5.80) | 1.20 (6.02) | 0.14 (4.13) |
|  | EMD (SE)  vs. PBO | 8.53 (7.09) | 2.47 (6.41) | 22.80 (8.32) | 18.36 (7.03) | 1.06 (6.99) |  |
|  | 95% CI | (-6.40, 23.45) | (-11.09, 16.04) | (5.34, 40.27) | (3.48, 33.25) | (-13.76, 15.89) |  |
|  | P | 0.25 | 0.70 | 0.01 | 0.02 | 0.88 |  |
| D3 | EM (SE) | 13.41 (5.12) | 2.61 (4.80) | 22.94 (6.39) | 23.50 (5.54) | -0.80 (5.76) | 3.94 (3.83) |
|  | EMD (SE) | 9.47 (6.76) | -1.33 (6.03) | 19.00 (7.99) | 19.56 (6.63) | -4.75 (6.60) |  |
|  | 95% CI | (-4.71, 23.65) | (-14.04, 11.37) | (2.26, 35.74) | (5.59, 33.52) | (-18.64, 9.15) |  |
|  | P | 0.18 | 0.83 | 0.03 | 0.01 | 0.48 |  |
| D4 | EM (SE) | 13.91 (5.46) | 3.11 (5.17) | 23.61 (6.76) | 26.17 (5.96) | 6.87 (6.17) | 2.94 (4.10) |
|  | EMD (SE)  vs. PBO | 10.97 (7.17) | 0.17 (6.49) | 20.66 (8.41) | 23.22 (7.14) | 3.92 (7.11) |  |
|  | 95% CI | (-4.05, 26.00) | (-13.50, 13.84) | (3.08, 38.25) | (8.20, 38.25) | (-11.05, 18.89) |  |
|  | P | 0.14 | 0.98 | 0.02 | 0.005 | 0.59 |  |
| D7 | EM (SE) | 22.16 (6.22) | 6.11 (5.96) | 34.28 (7.58) | 30.84 (6.87) | 8.87 (7.06) | 16.66 (4.67) |
|  | EMD (SE)  vs. PBO | 5.50 (8.08) | -10.55 (7.48) | 17.62 (9.38) | 14.18 (8.23) | -7.79 (8.20) |  |
|  | 95% CI | (-11.38, 22.39) | (-26.28, 5.18) | (-1.88, 37.12) | (-3.12, 31.48) | (-25.04, 9.45) |  |
|  | P | 0.50 | 0.18 | 0.07 | 0.10 | 0.35 |  |
| D14 | EM (SE) | 33.41 (7.20) | 13.86 (6.98) | 41.61 (8.66) | 35.50 (8.05) | 17.87 (8.21) | 18.75 (5.51) |
|  | EMD (SE)  vs. PBO | 14.66 (9.33) | -4.89 (8.81) | 22.85 (10.67) | 16.75 (9.68) | -0.89 (9.65) |  |
|  | 95% CI | (-4.85, 34.16) | (-23.41, 13.63) | (0.62, 45.09) | (-3.61, 37.10) | (-21.19, 19.41) |  |
|  | P | 0.13 | 0.59 | 0.04 | 0.10 | 0.93 |  |
| D28 | EM (SE) | 40.66 (8.07) | 19.36 (7.87) | 41.61 (9.63) | 52.50 (9.09) | 21.53 (9.23) | 31.23 (6.08) |
|  | EMD (SE)  vs. PBO | 9.43 (10.34) | -11.87 (9.88) | 10.38 (11.75) | 21.27 (10.87) | -9.70 (10.85) |  |
|  | 95% CI | (-12.18, 31.04) | (-32.63, 8.89) | (-14.10, 34.86) | (-1.57, 44.12) | (-32.50, 13.11) |  |
|  | P | 0.37 | 0.25 | 0.39 | 0.07 | 0.38 |  |

A post-hoc comparison between ALMB‑0166 and placebo was conducted for efficacy outcomes through Day 28 using a Mixed‑Model for Repeated Measures (MMRM). The MMRM included baseline score as a covariate, with visit, treatment group, and visit‑by‑treatment interaction as fixed effects, and was tested at a two‑sided α level of 0.05. D, Day. EM, Estimated mean. SE, Standard Error. EMD, Estimated mean difference. PBO, Placebo.
